# Pharmacopœia Collegii Regalis Medicorum Londinensis

**Published:** 1837-07

**Authors:** 


### Art. VII.

1. *Pharmacopœia Collegii Regalis Medicorum Londinensis.—Londini*, 1836. 8vo. pp. 208 and xxxii. 32mo. pp. 216 and xxxv.
2. *The Translation of the Pharmacopœia of the Royal College of Physicians of London*, 1836. *With Notes and Illustrations.* By Richard Phillips, F.R.S. L. & E. &c.; Lecturer on Chemistry at St. Thomas's Hospital. *By Permission.—London*, 1837. 8vo. pp. 392.
3. *The Pharmacopœia Collegii Regalis Medicorum Londinensis, translated, with a Commentary, chemical, pharmaceutical, and medicinal.* By D. Spillan, M.D., Fellow of the King and Queen's College of Physicians in Ireland.—*London*, 1837. 12mo. pp. 308.
4. *A Translation of the New Pharmacopœia of the Royal College of Physicians of London; with Notes and Criticisms.* By G. F. Collier, M.D., Member of the Royal College of Physicians, &c. &c.—*London*, 1837. Royal 8vo. pp. 272.

This work has been long and anxiously expected by the profession, and by many it has been thought to be too long delayed. The publication of a Pharmacopœia, however, by the highest authority, is a serious matter: it signalizes an era, not only in the ancillary sciences of botany and chemistry, but in the practice of physic itself; and we are therefore not among the foremost to complain, if the publication of a formulary, which must be the text-book of fifteen or twenty thousand practitioners, is accompanied rather by an excess than a deficiency of cautious delay. So rapid is now the advance of pharmaceutical and medical knowledge, that, while only three Pharmacopœiæ were published during the whole of the last century, namely, in 1720, 1745, and 1787, three have been already issued during the present, namely, in 1809, 1824, and 1836.*

The one before us, although not free from errors and imperfections, is not unworthy of the present state of science, or the character of the learned body from which it has proceeded. We cannot, however, but regret extremely that the design of the London College of compiling a Pharmacopœia in conjunction with the Colleges of Edinburgh and Dublin, was not accomplished. The negociations set on foot for this purpose were, it seems, begun, but were ultimately broken off, owing chiefly to the mutual distance of the parties. The advantages of a British or national Pharmacopœia are so obvious, that it is needless to dwell upon them here; but we trust that the time is not far distant when not only this object will be attained, but others of still greater importance, calculated to draw into closer union all the members of our common profession throughout the empire.

Mr. Phillips's Translation is exact, though in some places rather too literal; and the chemical notes which he has appended are excellent. At the request of the College, he "either conducted or inspected the preparation of almost every medicine which has been introduced, and in many cases repeated the processes which the Pharmacopœia already contained." (*Advertisement*, p. v.) The nature and object of his remarks are thus stated by himself:

"In preparing the remarks which accompany this Translation, my attention has been directed to two classes of persons:—First, those who may have been for some time engaged in the practice of physic, but who, not having watched the rapid progress which chemistry has made within a few years, are imperfectly acquainted with the important changes which it has produced by the introduction of new medicines from various and unexpected sources. The other class to which I allude is the numerous one of medical students: to these I have found, by no inconsiderable experience, that concise descriptions of the chemical changes which occur during the preparation of medicines have been extremely useful. They who know how small the portion of time is which the medical pupil has at his disposal for the acquirement of chemical

* We might have added the one of 1815, which, though commonly reputed a mere reprint of that of 1809, does in reality differ from it, as we shall afterwards have occasion to show; but then the same kind of improved reprints had probably occurred in the last century likewise: thus, Dr. Powell tells us that "it will explain some seeming inaccuracies in the references to the Pharmacopœia of 1787, if I mention that successive editions have varied somewhat from each other, and that unfortunately they have been confounded together. The quarto and the first duodecimo agree together; an octavo was afterwards published with alterations, and a duodecimo since with more alterations still, and these can only be distinguished by looking for some known point of difference between them in the body of the work." (Translation of the Pharmacopœia of 1809, second edition. *Preface*, p. xxviii.)

Mr. Phillips always calls it the edition of 1788, instead of 1787; and so does Gray, in his Supplement to the Pharmacopœia. (Fourth edit. 1828.)

and pharmaceutical knowledge, will readily admit the propriety of assisting his progress by familiar modes of illustration. With this latter view, I have made much use of diagrams." (*Adv.* p. v.)

We wish that, for the sake of students, Mr. Phillips had explained the notation of the chemical symbols which he has adopted from Berzelius, Brande, and Turner.

He has chiefly derived his account of the uses of medicines from Dr. Hue and Dr. Paris; but in this department he is too laconic, and it is to be regretted that those eminent physicians did not contribute to this part of the work on a scale proportioned to its utility. Mr. Phillips's book, however, is one of remarkable merit, and, like his previous ones on the same subject, will be a mine for all subsequent commentators on the Pharmacopœia.

Dr. Spillan's work constitutes a compendious Dispensatory, not devoid of merit, though, from the extreme haste with which it was brought out, it necessarily contains more errors than it would have done had the author allowed himself a reasonable time for its composition. There is more of chemistry and a good deal less of medicine in it than we anticipated. From a practical physician like Dr. Spillan, we might expect that the remarks on the therapeutical qualities and administration of the different medicines would be fuller than in the work of Mr. Phillips; and, generally speaking, this is the case; yet the difference is less than might have been looked for.

This gentleman seems unfortunate in the circumstances under which he undertakes his literary engagements. It is not long since we found it necessary to criticise his mode of rendering into his native tongue a foreign work of great importance, (See our Review of his Translation of Andral, Vol. I. p. 188;) and he has laid himself open to a like censure, although in a lesser degree, on the present occasion. In both cases, we believe, the defects in his performance have sprung from his having executed his task too hastily; and in neither case, we apprehend, will this be received by the profession as a sufficient excuse. When a medical writer condescends, from the importunities of publishers, or any other considerations extrinsic to science, to come before the public, without regard to the appearance he may make, he has certainly forfeited his claim to the indulgence of critics, at least, if not also of his readers generally.

Dr. Collier's book is on the same general plan, yet considerably different from the other two translations. It is much fuller in its therapeutical details, and much less explicit in its chemical explanations. The translation is correct, though not always very neat; and the pharmaceutical and practical additions are for the most part sensible, and bear the stamp of experience. The volume, however, is greatly disfigured by the general style of the criticisms contained in it. These are throughout marked by an angry and bitter tone, which, to say the least of it, is ungraceful and undignified, and assuredly exposes the author as much as the censors of the College. The advertisement of the original, and its authorized version by Mr. Phillips, was accompanied by an announcement that the College would not permit any other translation. To us it seemed probable that this mistaken notice was to be attributed rather to the publisher than to the College. Dr. Collier, however, thought otherwise, and put forth a facetious pamphlet, in which he overwhelms that learned body with indignant merriment; and not only reproaches them with this their mistake touching the true construction of the Copyright Act, but with all and singular their mistakes from the beginning of time. These subjects, though discussed, were by no means exhausted in the pamphlet, and they are resumed in the work before us. The author tells us, that "he resents the injury the College have inflicted on one *who has certain claims on their consideration, which will be understood as well by their fellows as by their licentiates*," (p. iv.;) and he concludes his preface with these words "To the Censors he has merely to say, 'Gentlemen, behold the book!' Now, therefore, 'resist,'—or recant, or retreat,—with what silent dignity you may!"

We know not exactly what claims Dr Collier may have on the consideration of the London College, nor the extent of the provocation he has received from it: we do not even deny that many of his criticisms are just in point of fact: it is on the ground of taste and propriety that we blame him, for loading the pages of an elementary didactic work with taunts, reproofs, and recriminations, totally at variance with the character of such a work. When neither too brief nor too wrathful, Dr. Collier is always instructive; and if he will, in his next edition, forego his resentment, and fill up his columns with additional practical matter; and if, at the same time, he lessens his type and page, and consequently his price, we will promise to his work every success. We would also advise him to introduce, like Mr. Phillips, all the old synonymes, a thing absolutely necessary to keep the older practitioners in the right path.

Both Mr. Phillips and Dr. Collier have added a table of doses, which will be useful to young practitioners, many of whom will wish that they had accentuated the words, and thus given the *quantities* in a double sense. We might find much to say upon this table, by comparing the doses with those given by authors who have extolled a favorite remedy, as in the cases of tartar emetic, carbonate of iron, and alum; but to every thinking reader such reflections will be sufficiently obvious.

In executing our present task, we are not disposed to follow the example of Dr. Collier, although there are not a few faults in the Pharmacopœia, both of omission and commission, which are very open to criticism. At another time, perhaps, we may advert to these in detail; on this occasion, we wish to confine ourselves chiefly to the making our readers acquainted with the Pharmacopœia such as it is, and with the principal alterations that have been made in it. We shall, however, as we go along, notice such of the commentaries of the different translators as relate to the action and use of new or important remedies, with the view of giving the article as practical a character as possible.

*Weights and Measures.* Instead of the wine gallon, the imperial is now to be used; so that the gallon and the pint will be larger, in the proportion of about five to four, (or, more exactly, in the proportion of 277.274 to 231;) but, as the College have now divided the pint into twenty ounces instead of sixteen, the ounce, drachm, and minim will be the same as before, within an insignificant fraction.

After observing, as formerly, that acids, alkalies, &c. should be kept in stoppered glass bottles, they add, that some preparations require the bottles to be black or green.

The saturation of alkalies or acids is to be tested by litmus and turmeric: this improvement is introduced into the formula for Liquor Ammoniæ Acetatis.

Specific gravities are to be taken at 62° instead of 55° of Fahrenheit. Crucibles are to be either Hessian or Cornish.

*Nomenclature.* The alterations of nomenclature are numerous, but in many cases inevitable. The College having once adopted the plan of giving names supposed to express the chemical nature of substances, it is impossible to retain them when the names have been demonstrated to be erroneous; and it is therefore obvious that such names as Hydrargyri Submurias, Hydrargyri Oxymurias, &c. must be expunged: but we agree with Hufeland, and we believe with the majority of practitioners, that for practical purposes it would be far better to retain the old names, as calomel, corrosive sublimate, &c., which involve no chemical theory, and are therefore not so fleeting as the scientific appellations. For the benefit of those who wish to study chemistry, a Pharmacopœia should contain the synonymes of the day, but, for the benefit of practical men, it should contain permanent names.

Something similar might be said of the botanical appellations, where, from the perpetual splitting of genera, no name is safe. There is an additional inconvenience attending this shifting nomenclature, as it occasions trouble, and even doubt, to the reader, whenever he turns to the authors of a period wherein names were used which a new Pharmacopœia has made obsolete.

*Materia Medica.* In the names of plants, the College chiefly follow Linnæus, Willdenow, and De Candolle, though Don, Smith, Sprengel, and others are often referred to; in those of animal substances, their authority is Cuvier; in chemical names, they follow recent writers, without selecting any one in particular.

The following articles are new; that is, are not in the Materia Medica of the last Pharmacopœia.

Aconiti radix; Ammoniæ liquor fortior, (specific gravity .882;) Aurantii flores; Aurantii oleum; Barytæ carbonas; Bergamii oleum, (oil of bergamot;) Brominium; Calcis hydras, (slacked lime;) Calx; Carbo animalis; Chimaphila, (winter-green, or pyrola;) Creasoton; Curcuma, (turmeric;) Diosma, (buchu;) Ergota; Ferri Percyanidum, (percyanide of iron, or Prussian blue;) Hirudo; Iodinium; Lacmus, (litmus;) Lactucarium; Limonum succus; Lini oleum; Lobelia; Manganesii binoxydum; Maranta, (arrow-root;) Nux vomica; Pareira; Phosphorus; Potassæ chloras; Potassi ferrocyanidum; Quina; Sabadilla; Sago; Sodæ acetas; Sodæ phosphas; Sodii chloridum; Spiritus vini Gallici, (brandy;) Vinum Xericum, (sherry.)

We shall confine ourselves to a few observations on these substances.

*The root of aconite* is used in making aconitina.

*The stronger solution of ammonia* contains about thirty per cent. of ammonia, and is therefore three times as strong as the ordinary solution; it may consequently be reduced to the strength of the Liquor ammoniæ by mixing it with a double quantity of distilled water. In the passage where this fact is stated, there is an erratum of *tribus* for *duabus*, at p. 29 of the 8vo, and p. 34 of the 32mo edition. These misprints are not mentioned in the tables of errata. Mr. Phillips has, in like manner,

"three" for "two," (p. 25,) but has given the latter in his list of errata. Dr. Spillan has noticed the mistake in a note to his translation, p. 24; Dr. Collier has not. For a table showing the weight of pure ammonia contained in one hundred parts of the solution, at different specific gravities, the reader may consult Sir H. Davy's Elements of Chemical Philosophy, p. 268, or Brande's Manual of Pharmacy, p. 215.

*Chimaphila* leaves are diuretic; and a decoction of them is among the preparations.

*Lactucarium* is the inspissated juice of the *Lactuca sativa*, and very nearly resembles, if it is not identical with the *Extractum Lactucæ*, which afterwards occurs among the preparations. We believe the chief difference between the two preparations is, that the original Lactucarium of Dr. Duncan is the inspissated juice collected from the growing plant, by cutting off the heads of the plant when in flower; while the College extract is formed from the juice expressed from the pounded leaves. The former is the stronger narcotic; Dr. Collier stating its dose to be from gr. ij. to gr. iv., while that of the extract he fixes at from gr. v. to ℈j.

*Pareira* root is assigned to the *Cissampelos Pareira*, on the authority of De Candolle; it is more commonly known by the name of *Pareira brava*. It has been of late years much used by surgeons; and has, in particular, been highly extolled by Sir B. Brodie, who, after observing that he has been disappointed in the use of the uva ursi in chronic inflammation of the bladder, says, "I have seen much more good done by a very old medicine, which has been long ignominiously but unjustly expelled from the Pharmacopœia of the College of Physicians, namely, the root of the Pareira brava; and, with regard to this, I am satisfied that it has a great influence over the disease which is now under our consideration, lessening very materially the secretion of the ropy mucus, which is in itself a very great evil, and, I believe, diminishing the inflammation and irritability of the bladder also." (*Lectures on the Diseases of the Urinary Organs*, p. 88-9.)

*Sabadilla* (or Cevadilla) seeds are assigned to the *Helonias officinalis*, on the authority of Don: veratria is procured from them.

The following substances in the Materia Medica of the last Pharmacopœia are omitted in that of the present one:—Acetosæ folia; Acidum aceticum fortius; Acidum citricum; Bistortæ radix; Hellebori fœtidi folia; Lactuca; Linum catharticum; Magnesiæ subcarbonas; Rubiæ radix; Salicis cortex; Spongia; Tartarum.

The alterations in the nomenclature, &c. of the Materia Medica are very numerous; the following list contains the greater part of them.

We may preface it by observing, that a very brief method of designating the articles has been adopted in the left-hand column of the list, as well as in the body of the work. Thus, *Senna* stands for senna leaves, *Adeps* for Adeps præparatus, *Uva* for stoned raisins, &c.; but, as the meaning of the left-hand column is always defined in the right, brevity has been gained without obscurity.

*Acidum Sulphuricum* is directed to be of the specific gravity 1.845, instead of 1.850.

*Alumen* is called a sulphate, instead of a supersulphate, of alumina and potass.

*Ammoniacum* is assigned to the *Dorema Ammoniacum,* instead of the *Heracleum gummiferum.*

*Antimonii sesquisulphuretum* stands instead of *Antimonii sulphuretum.*

*Aurantii cortex* is assigned to the *Citrus vulgaris,* instead of the *Citrus Aurantium;* but the fruit is put down, as formerly, to the *Citrus Aurantium;* after which the word *Hispalense* is omitted.

*Balsamum Peruvianum* and *Bals. Tolutanum* are both ascribed to the *Myroxylon peruiferum;* the former being the liquid, and the latter the concrete balsam: the latter was ascribed in the last Pharmacopœia to the *Toluifera Balsamum.*

*Borax* is called a biborate, instead of a subborate of soda.

*Cardamomum* is put down to the *Alpinia Cardamomum,* instead of the *Matonia Cardamomum.*

*Caryophyllus* is ascribed to the *Caryophyllus aromaticus,* instead of the *Eugenia caryophyllata.*

*Castoreum* is said to be a *concretum in folliculis præputii repertum,* instead of a *concretum sui generis.*

*Centaurium* is assigned to the *Erythræa Centaurium,* instead of the *Chironia Centaurium.*

*Cetraria islandica* has replaced the *Lichen Islandicus.*

*Cinchona* is divided into the same species as before, but Lambert is referred to as the authority, instead of Zea.

*Copaiba* is assigned to the *Copaifera Langsdorfii,* instead of the *Copaifera officinalis.*

*Crocus* is no longer necessarily *Anglicus,* and the stigmata are directed to be dried.

*Cusparia,* formerly assigned to the *Cusparia febrifuga,* on the authority of Bonpland, is now set down to the *Galipea Cusparia,* in accordance with De Candolle.

*Cydonia* is assigned to the *Cydonia vulgaris,* instead of the *Pyrus Cydonia.*

*Fici* stands instead of *Caricæ fructus.*

*Fœniculum* is put down to the *Fœniculum vulgare,* instead of the *Anethum Fœniculum.*

*Galbanum* is assigned to the *Galbanum officinale,* instead of the *Bubon Galbanum.*

*Gallæ* were formerly defined to be the nest of the *Cynips Quercûs folii;* but now are asserted to be the diseased buds of the *Quercus infectoria.*

*Helleborus* (the root of black hellebore,) is set down to the *Helleborus officinalis,* instead of *niger.*

*Jalapa* is ascribed to the *Ipomœa Jalapa,* instead of the *Convolvulus Jalapa.* This is stated to be on the authority of a MS. of Mr. Don; but we believe that jalap has been called the produce of an *Ipomœa* for some time. Thus, the late Professor Burnett says, "*Ipomœa* (or *Convolvulus*) *Jalapa* yields that well-known and useful purgative which bears, with the plant from which it is procured, a common specific name." (*Outlines of Botany,* p. 1003.)

*Ipecacuanha* is ascribed to the *Cephaelis Ipecacuanha,* instead of the *Callicocca Ipecacuanha.*

*Kino* is ascribed to the *Pterocarpus erinaceus,* instead of the *Pterocarpus erinacea.*

*Limones* are ascribed to the *Citrus Limonum*, instead of the *Citrus Medica*.

*Manna* is assigned to the *Ornus europæa*, instead of the *fraxinus Ornus*.

*Moschus* is called *Humor in folliculo præputii secretus*, instead of *Concretum sui generis*.

*Mucuna* stands for *Dolichos*, (cowhage.)

*Myrrha*, formerly called *Arboris nondum descriptæ gummi-resina*, is asserted to be the gum-resin of the *Balsamodendron Myrrha*, on the authority of Ehrenberg.

*Olibanum* is said to be derived from the *Boswellia serrata*, instead of the *Juniperus Lycia*.

*Opium* is no longer necessarily Turkish.

*Opopanax* is said to be derived from the *Opopanax Chironium*, instead of the *Pastinaca Opopanax*.

*Saccharum* formerly meant brown sugar, and *saccharum purificatum* the refined; but now *Sacchari fæx* is used for treacle, and *Saccharum* for refined sugar.

*Sagapenum*, formerly called *plantæ nondum descriptæ gummi-resina*, is now said to be the gum-resin of a *Ferulæ species incerta*.

*Sapo* now stands for *Sapo durus*, and is no longer limited to the Spanish kind.

*Sarsaparilla* is now called *Sarza*, and is assigned to the *Smilax officinalis*, instead of the *Smilax Sarsaparilla*.

*Sassafras*. The root only is now officinal, the wood being omitted.

*Scammonium* (the gum-resin,) stands for *Scammonea:* the name of the plant is still *Scammonea*.

*Scoparius*, set down to the *Cytisus Scoparius*, stands for *Spartii cacumina*, formerly set down to the *Spartium scoparium*. The tops are ordered to be fresh: it was not directed what state they were to be in before.

*Senna*, formerly set down to the *Cassia Senna*, is now assigned both to the *Cassia lanceolata* and the *C. obovata*.

*Simaruba* (instead of *Simarouba*,) is assigned to the *Simaruba officinalis*, instead of the *Quassia Simarouba;* and the bark of the root is directed to be used, instead of simply the bark.

*Spiritus rectificatus* is directed to be of the specific gravity .838, instead of .835.

*Spiritus tenuior* is directed to be of the specific gravity .920, instead of .930, so as to be of the strength prescribed by Act of Parliament for proof spirit.

*Stannum* no longer has the definition *Stanni limatura* in the right-hand column.

*Sulphur* is now defined to be *Sulphur sublimatum:* formerly both substances stood in the left-hand column.

*Tabacum* is no longer limited to the Virginian sort.

*Tormentilla* is assigned to the *Potentilla Tormentilla*, instead of the *Tormentilla officinalis*.

*Tragacantha* is called a concrete juice, instead of a gum.

*Uva ursi* is assigned to the *Arctostaphylos Uva ursi*, instead of the *Arbutus Uva ursi*.

*Zingiber* is made of the feminine instead of the neuter gender.

In the list of the Materia Medica, as well as throughout the work, *di* stands instead of *sub* in the names of the sub-salts; as, *diacetas* instead of *subacetas*. Many of the botanical terms have been reformed: thus, the *cormus* stands instead of the *bulbus* of colchicum, the *bulbus* instead of the *radix* of *allium*, the *fructus* instead of the *baccæ* of *aurantium*, and the *rhizoma* instead of the *radix* of *zingiber*. In the names of plants, gentile adjectives are now made to begin with a small letter; as, *Ornus europæa*, *Spigelia marilandica*.

The part of the work which we have just noticed is followed by a section which is entirely new, and extremely useful: it consists of notes, chiefly on the chemical preparations, by which their purity may be ascertained. To the translations of these tests the authors have added explanatory remarks, which will be a treasure to students. The following is a specimen:

"ACIDUM HYDROCYANICUM DILUTUM. *Dilute Hydrocyanic Acid.*—Free from colour; goes off in vapour by heat, exhaling its peculiar odour. It turns litmus of a light fugacious red colour; hydrosulphuric acid, when added, does not discolour it. One hundred grains of this acid, when solution of nitrate of silver is added, precipitate 10 grains of cyanide of silver, which are readily dissolved by boiling nitric acid. If the iodo-cyanide of potassium and mercury, when mixed with the hydrocyanic acid, is reddened, it contains some other acid. In 100 grains of this diluted acid, there are contained 2 grains of real hydrocyanic acid; and to this standard, in whatever mode it is distilled, we direct it should be reduced.

" *Remarks.* Its total evaporation shows the absence of fixed impurity. If the hydrocyanic acid redden litmus paper strongly and permanently, then some other acid is mixed with it: the absence of most metallic salts is denoted by the non-action of hydrosulphuric acid. If the hydrocyanic acid contain hydrochloric acid, then the precipitate formed by nitrate of silver, being chloride of silver, is insoluble in the nitric acid. Any acid mixed with the hydrocyanic acid decomposes the iodo-cyanide of potassium and mercury, and forms biniodide of mercury, which is of a red colour." (*Phillips*, p. 22.)

The reason does not appear clear why the mode of determining the strength of hydrocyanic acid by its specific gravity should not have been adopted, as in the case of all the other liquid acids. We believe it to be susceptible of equal, if not greater accuracy than the one directed, and it is certainly less tedious and complicated.

Dr. Collier seems to entertain no very high opinion of the therapeutic value of this medicine. " It is commonly used for suicide, (he says,) and is fit for little else, with the exception of its use externally as a lotion in some cutaneous diseases, attended with intolerable pain and itching, and not extended over too large a surface." (P. 54.) We agree with him so far as to believe that few remedies are more frequently prescribed without obvious effect. Surely, however, Dr. Collier's definition of the dose,—"internally, from five minims upwards,"—is not sufficiently close for so dangerous a remedy: it is adding the risk of chance-medley to suicide.

Under the head of Aconitina, the very proper caution is given, "*viribus hæc induta violentis, non temerè adhibenda est.*" Similar advice is given under the heads *Strychnia* and *Veratria*. As it is the office of a Pharmacopœia rather to indicate established remedies, than those which are yet struggling for existence, we think it would have been as well if the first and last of these three potent alkaloids had been omitted: the practitioners who have confidence in them might still have prescribed them, without their being necessarily kept by every druggist in England.

The *Præparata et Composita* are arranged in alphabetical order; as the *Acida*, *Ætherea*, *Alkalina*, &c.

*Acida.* Under this head we find five new preparations; namely, *Acidum Aceticum*, *Acetum Cantharidis*, *Acidum Hydrochloricum dilutum*, and *Acidum Phosphoricum dilutum.* The acetic acid is made by decomposing the acetate of soda with sulphuric acid, by which a purer, but we fear a much dearer, acid will be produced than the pyroligneous acid of the last Pharmacopœia. The *Acid. Hydrochl. dil.* (dilute muriatic acid,) is formed by mixing one part of the strong acid with three of distilled water, and is rather stronger than dilute sulphuric acid; for,

"One fluidrachm of the under-mentioned diluted Acids saturates, very nearly, the annexed quantity of crystallized Carbonate of Soda:

| | |
|---|---|
| "Acidum Hydrochloricum dilutum . . | 32 grains. |
| Acidum Sulphuricum dilutum . . | 28 — |
| Acidum Nitricum dilutum . . . . | 19 —." |

(*Phillips's Translation*, p. 62.)

The dose of the dilute phosphoric acid is stated by Mr. Phillips to be from ♏xx. to f.ʒj.; by Dr. Spillan, from ♏xx. to ʒss.; and by Dr. Collier, from ♏xxv. to ʒj. Lobstein gave it, in phthisis, in the dose of twenty or thirty drops in a glass of *eau sucrée* made with distilled water, every three hours. It was necessary that the case should be free from any inflammatory complication, and the patient drank sugared milk after every dose. (*Formulaire de Magendie*, 8e Edit. p. 392.)

The *Acidum Aceticum dilutum* of the late Pharmacopœia is now more conveniently called *Acetum destillatum.*

An important improvement has been effected in the process for making tartaric acid. Formerly, the bitartrate of potash gave up only its excess of acid; but now the remaining tartrate of potash is also decomposed.

"Take of Bitartrate of Potash four pounds,
Boiling distilled Water two gallons and a half,
Prepared Chalk twenty-five ounces and six drachms,
Diluted Sulphuric Acid seven pints and seventeen fluidounces,
Hydrochloric Acid twenty-six fluidounces and a half, or as much as may be sufficient.

"Boil the bitartrate of potash with two gallons of distilled water, and add gradually half the prepared chalk; afterwards, the effervescence having ceased, add the remainder of the chalk, first dissolved in the hydrochloric acid with four pints of distilled water. Lastly, set by, that the tartrate of lime may subside; pour off the liquor, and wash the tartrate of lime frequently with distilled water, till it is free from taste. Then pour on it the diluted sulphuric acid, and boil for a quarter of an hour. Evaporate the strained liquor with a gentle heat, that crystals may be formed.

"Dissolve the crystals, that they may be pure, again and a third time in water, and strain it as often; boil down, and set it aside." (*Phillips's Translation*, p. 78.)

*Ætherea.* The late Pharmacopœia contained an *Æther sulphuricus*, and also an *Æther rectificatus*; the former, to quote the words of Mr. Brande, "being a sulphurous mixture of æther, alcohol, and water, probably never intended to be prescribed, and certainly quite unfit for medicinal use; but, in chemical language, sulphuric æther and rectified æther are synonymous; and if the physician prescribes the former instead of the latter, (a mistake which frequently happens,) it is probable that the patient may get the heterogeneous compound just adverted to, instead of the pure æther which was intended." (*Manual of Pharmacy*, p. 455-6.)

The possibility of error has now been taken away, for the purified preparation alone remains, and bears the name of *Æther sulphuricus*. The *Spiritus ætheris aromaticus* and the *Spiritus ætheris sulphurici* have also been omitted: the method of making the *Oleum æthereum* has been improved.

*Alkalina.* Under this section we have the following new articles:—*Aconitina*, *Morphia*, *Morphiæ acetas*, *Morphiæ hydrochloras*, *Quinæ disulphas*, *Strychnia*, and *Veratria*. The *Ammoniæ subcarbonas* is now called *Ammoniæ sesquicarbonas*.

The following observations of Dr. Collier on the new preparations of opium are interesting.

"The superiority of acetate and muriate of morphia over the old preparations of opium has been explained by the supposition of its acting as an anodyne without producing the subsequent derangement of the brain attended with headach, vertigo, sickness, and febrile symptoms, of which patients so often complain, and these last effects (without much evidence) have been ascribed to the narcotin of the drug." . . . "Long before the discovery of morphia, it had been noticed, that certain acidulated preparations of opium produce the anodyne effects of that drug without the subsequent disturbance of the brain and stomach. Hence, the reputation of the Sunderland drop, the black drop of Braithwaite, and several preparations made with lemon-juice. Then came the discovery of the alkali; and we were told, that the stupifying power resides in the narcotine, and the sedative or anodyne power in the alkaline. Now, Bally asserts, that narcotine has little or no action on the human body. Orfila considers it stupifying and deleterious; while Magendie deems it a powerful excitant. Subsequent discoveries have shewed (whether as products or educts is a matter of doubt) that opium, besides meconic acid and morphia, contains five alkaloids, viz. codeia, narceia, meconia, thebaia, and narcotina. Their medicinal action, indeed, has not yet been determined, but the complexity of the constitution of the drug will perhaps account for the difference of opinion on the medicinal properties of the narcotine. The Editor has observed, that with opium-eaters the salts of morphia are not adequate substitutes, at least, as far as his observation has extended; and this renders it probable, that the stimulant properties reside either in the narcotine or in some other principle. As new wine will produce headach, so will new opium; but age (if we may trust to the experience of the habitual consumers) equally improves the drug, and corrects that stupifying and pernicious quality which so often embarrasses the head and stomach. If this view of the salts of morphia be correct, they must be inefficient for all stimulant indications; inefficient to support the powers of life in variola confluens, and in typhoid diseases; inefficient in the cold stages of ague to bring on the hot; and inefficient to support the vigour of the circulation in abscesses. On the other hand, they will be more applicable where anodyne effects are alone aimed at." . . . "This salt [the hydrochlorate] is uniform in its constitution; but the acetate of morphia varies. Besides it has another advantage over the acetate, in not being deliquescent; whereas the neutral acetate is decomposed by water." (P. 63-4.)

Dose of Acetate, from gr. $\frac{1}{10}$ to gr. j.; of Hydrochlorate, gr. $\frac{1}{6}$ to gr. ij. (Collier.)

*Animalia.* This section contains one new substance, *Carbo animalis purificatus*. *Spongia usta* has been omitted.

*Aquæ destillatæ.* Two new waters have been added; namely, *Aqua florum Aurantii* and *Aqua Sambuci*. In noticing the preparation of the latter, Dr. Collier asks, "Where is the oil to be obtained? They do not mean the green oil?"—forgetting that the particular oil to be used (viz. that distilled from elder-flowers,) is given among the Olea destillata.

*Cataplasmata.* Here are two additions, the *Cataplasma Conii,* and the *Cataplasma Lini.* On the former of these, which is directed to be made with two ounces of Extract of Hemlock, Dr. Collier very properly asks, "How is a poor man to pay for such a poultice, requiring to be renewed twice a day?" He recommends as a substitute four ounces of hemlock leaves boiled in half an ounce of lard, of which a portion is to be spread over a common poultice.

*Cerata.* A *Ceratum Hydrargyri compositum* has been added. The *Ceratum simplex* is now called merely *Ceratum.* In directing the method of making Ceratum Saponis, the College say, "℞. Saponis uncias decem," on which Dr. Collier asserts, in his "Cautions," that "hard soap is meant, but selection from a variety is left to the dispenser." (P. 80.)—Not so; for sapo is defined in the Materia Medica as *ex Olivæ oleo et Sodâ confectus.*

*Confectiones.* Water was formerly used in preparing the *Conf. Aromatica,* syrup in preparing the *Conf. Opii* and *Conf. Scammonii,* and honey with the *Conf. Piperis nigri* and *Conf. Rutæ;* but, in the first formation of these conserves, the dry ingredients only are now to be used, and the water, syrup, or honey added when the conserve is wanted for use. This is a great improvement, as the conserves prepared in the old way often become mouldy. In his remarks on the *Conf. Amygdalæ,* Mr. Phillips says, that "it is now very advantageously directed that the ingredients should be kept ready mixed in a dry state, and the water added to them when the confection is wanted for use." (P. 128.) No water, however, is directed to be added to them.

*Decocta.* The majority of the decoctions are now directed to be made with distilled water. The following are new:—*Decoct. Chimaphilæ, Decoct. Granati, Decoct. Scoparii comp., Decoct. Tormentillæ, Decoct. Uvæ ursi.*

The *Decoct. Chimaphilæ,* as formerly stated, is a diuretic. Dr. Collier says it is also slightly astringent, and notices the fact of its great reputation among the American Indians.

The *Decoctum Granati* is ordered to be made from the *bark of the fruit,* and the preparation is stated, both by Mr. Phillips and Dr. Spillan, to be an efficacious remedy in *tapeworm,* as well as dysentery. Now, we find, in all the best accounts published of the use of the pomegranate, that it is the *bark of the root,* not of the fruit, that has been found effectual in tænia. This is the part mentioned by Celsus, ("Mali Punici tenues radiculas." Lib. iv. c. 17.) Dr. Gomez, of Portugal, recommends this also; and in the communication of Dr. Buchanan, in the Edinburgh Medical Journal for January, 1807, "the fresh rind of the pomegranate root" is given as the Indian remedy for tapeworm. In Mr. Breton's paper on the same subject, in the 11th volume of the Medico-Chirurgical Transactions, the bark of the *root* is alone mentioned, as the remedy so successfully employed by him; and, in a recent communication from Dr. Leslie, in the Medical Gazette, (March 18, 1837,) we find it stated that the *bark of the fruit,* "which is known to be useful in chronic dysentery and diarrhœa, *is not of the slightest use in tapeworm.*" Our readers must therefore recollect that, in prescribing the officinal preparation of the Pharmacopœia, they are ordering the Indian remedy for chronic dysentery and diarrhœa, but not for tænia.

The strength of the decoction of the *Pomegranate-root-bark*, as used for tænia, has been various. The native Indian prescription, as given by Drs. Buchanan and Leslie, is ℥viij. of the bark to Oiij. of water, and boiled down to Oij.; the dose, a wine-glassful repeated at intervals. Mr. Breton employed ℥ij. of the root to Ojss., and boiled down to O½, and gave a wine-glassful every half-hour. The following is the formula contained in Radius's excellent *Heilformeln* for 1836:—℞. Cort. Rad. Granati rect. ℥ij.—℥iij. Macera in Aquæ lb. ij. per xxiv. horas, deinde coque ad rem. lb. j. Filtra, et adde Sacch. alb. q. s. ad grat. saporem. —*Dose*, the third part every half-hour.

Dr. Collier notices the preferableness of the root in cases of tænia, and recommends it from his own experience. His formula is, two ounces of the bark of the root in a pint of water, boiled down to half a pint, and he recommends the whole to be given in a couple of doses, after an interval of two hours.

The *Decoct. Scoparii comp.* is a very useful diuretic, made with broom-tops, juniper berries, and dandelion root; the *Sp. Armoraciæ comp.* and *Sp. Juniperi comp.* will be good adjuvants.

The *Decoct. Amyli* is the old *Mucilago Amyli;* and the *Decoct. Cetrariæ* is the old *Decoct. Lichenis*, reduced to half its former strength.

The *Decoct. Cinchonæ*, formerly made with the *Cinch. lancif.*, is now made with the *Cinch. cordif.*, with the observation appended, that the decoctions of the *Cinch. lancif.* and the *oblongif.* are made in the same manner. Our readers will recollect that the *Cinch. cordifolia*, or yellow bark, contains the greatest quantity of quina.

*Emplastra.* The *Empl. Belladonnæ* is new; the *Empl. Cumini* has been omitted.

The following observations of Dr. Spillan on the use of the *Emplastrum Cantharidis* are sensible, and merit attention.

"It should be observed, that, if in spreading the plaster, too hot an iron be employed, the efficacy of the cantharides is considerably diminished. Blisters are commonly allowed to remain on much longer than is necessary—this happens principally in the case of children—and many serious accidents have occurred in consequence. Blisters should never be allowed to remain longer on children than three, or at most four, hours; they should then be removed, even though vesication may not appear to have taken place. Very ugly sores have been occasioned by neglect of this precaution. Where ulceration has been produced by blisters, an emollient cataplasm is the best application that can be made. Where strangury occurs, diluent drinks should be indulged in pretty freely. Before the blister is applied, it is found of use to wash the part in warm vinegar. With respect to the therapeutical effects of blisters, it may be well to observe, that they are intended to act as derivatives or revulsives; and it is no harm to remind the junior practitioner that they never should be employed whilst the fever accompanying internal inflammation is high; they will only serve to exasperate all the symptoms. In fact, the employment of blisters requires, on the part of the practitioner, very serious consideration, though there is no therapeutic agent resorted to among routine practitioners with more *sang froid* than blistering." (P. 102.)

On another preparation of cantharides, however, now for the first time introduced into the Pharmacopœia, the Acetum Cantharidis, surely the laconic observation "USE—stimulant and diuretic," is defective if not erroneous. Mr. Phillips very properly states that this formula "is employed as an extemporaneous blister;" and Dr. Collier's definition is still more explicit: "A prompt form for counter-irritation, long anticipated by provincial and domestic recipes, as well as by the common practice of fomenting with hot vinegar before the application of a blister." (P. 48.) Dr. Spillan's omitting to notice this, its most legitimate application, is the more remarkable, as it is specifically indicated in the Pharmacopœia by the term *Epispasticum*, subjoined in parenthesis.

*Enemata.* These are all new: they are the *Enema Aloës, E. Colocynthidis, E. Opii, E. Tabaci,* and *E. Terebinthinæ.* Perhaps we ought to have excepted the *Enema Tabaci*, which is the *Infusum Tabaci* removed to this place. It is weaker in the proportion of four to five; being now made with a drachm of tobacco to twenty instead of sixteen ounces of water.

Dr. Spillan says, the *Enema Tabaci* "has been applied as a stimulus to the rectum;" and Mr. Phillips calls it "a very drastic enema, recommended by some in cases of hernia, but with doubtful success." (P. 153.) Dr. Collier very properly calls it "violently depressing and relaxing," it being neither stimulant nor drastic. Its use, as is well known to practitioners, is by no means limited to cases of hernia. Dr. Abercrombie has the highest confidence in its powers in ileus and enteritis; but he begins with a weaker form than the one of our Pharmacopœia, "perhaps fifteen grains infused for ten minutes in six ounces of boiling water: after the interval of an hour, if no effect has been produced, it may be repeated in the quantity of twenty grains, and so on, until such effects are produced, in slight giddiness and muscular relaxation, as show that its peculiar action is taking place upon the system." (*On Diseases of the Stomach, &c.*, 2d Edit. p. 159-60.) We prefer this cautious employment of a remedy, of which the immediate effects are often alarming. Dr. Collier says, "Liquor of Ammoniæ, with brandy and water, is the best antidote, if the enema produces dangerous depression." (P. 100.)

*Extracta.* To this section have been added the *Extr. Colchici Cormi, Extr. Colchici aceticum, Extr. Digitalis, Extr. Pareiræ,* and *Extr. Uvæ ursi.* The resinous extract of cinchona has been thrown out, and the watery extract is directed to be made with the *Cinch. cordif.* instead of the *lancifolia;* but with the observation added, that extracts of the *Cinch. lancif.* and *oblongif.* are made in the same way.

*Infusa.* All the infusions are to be made with distilled water. The following have been added to the list:—*Inf. Diosmæ, Inf. Krameriæ,* (rhatany,) *Inf. Lupuli, Inf. Pareiræ, Inf. Scoparii, Inf. Serpentariæ, Inf. Valerianæ.* The *Inf. Cinchonæ* is still made with the lance-leaved bark, but the time of maceration is increased from two hours to six. The *Inf. Digitalis* is much weaker; a drachm of digitalis being used to twenty-one ounces of fluid, instead of eight and a half. On this Dr. Collier interposes his "Caution" as follows: "Mark! Whether by error or design, the College has ordered a drachm to a pint instead of a drachm to half a pint, the old standard. When the practitioner orders the infusion, what are you to do?" If the physician is still calculating upon the old infusion, and the druggist sends, as he ought to do, the new one, the mistake is happily on the safe side; but, if the druggist, in spite of his Majesty's proclamation, persists in making the infusion of the old strength, while the physician intends the new, the consequences might be less pleasant.

The present *Inf. Gentianæ comp.* is stronger than the former one, in the proportion of six to five.

The *Inf. Rosæ comp.* is now made with a greater proportion of rose-leaves, and the time of maceration is lengthened from half an hour to six hours.

*Linimenta.* The *Linimentum Opii* has been added; the *Linim. Terebinthinæ* altered, by substituting soft soap and camphor for the resin cerate; and the *Linim. Saponis C.* is now called simply *Linim. Saponis.*

*Mellita.* Oxymel is now made with a pint and a half of strong acetic acid to ten pounds of honey, instead of a pint of distilled vinegar to two pounds of honey; and, instead of the long evaporation formerly required, the honey is now merely heated, and the acid mixed with it.

On this preparation Dr. Collier remarks, in his peculiar style, "You ought not to use this in dispensing, without a distinct understanding with the prescriber of it. The editor has taken the trouble to prepare it, and, finding that the College translator prescribes the dose from a fluidrachm to a fluidounce, feels it his duty to remark, that it is not a fluid in such proportions, and that in such a dose it is so powerful that it would induce spasmodic cough, gripes, &c. The censors ought to be indicted, under Lord Ellenborough's Act, for cutting throats." (P. 127.)

*Metallica.* The following articles in this section are new:—*Argenti Cyanidum, Liquor Argenti Nitratis, Barii Chloridum, Liquor Barii Chloridi, Calx chlorinata, Ferri Iodidum, Hydrargyri Bicyanidum, Hydrargyri Iodidum, Hydrargyri Biniodidum, Plumbi Chloridum,* (used in preparing the hydrochlorate of morphia,) *Plumbi Iodidum, Plumbi Oxydum hydratum,* (used in preparing the disulphate of quina,) *Liquor Potassæ effervescens,* (potash-water,) *Potassii Bromidum, Potassii Iodidum,* (hydriodate of potash,) *Liquor Potassii Iodidi comp., Liquor Sodæ effervescens,* (soda-water,) *Liquor Sodæ chlorinatæ.*

The *Liquor Ferri alkalinus, Vinum Ferri,* and *Hydrargyrum purificatum* are omitted.

The *Antimonii sulphuretum* is now called *Antimonii oxysulphuretum. Antimonium tartarizatum* is now called *Antimonii Potassio-tartras,* and the mode of preparation is altered. Mr. Phillips observes, that "in the last Pharmacopœia *glass of antimony* was used in preparing this medicine: it is, however, not only difficult to obtain it, but glass of lead is frequently substituted for, and, what is still worse, mixed with it." (P. 190.) Mr. Phillips has omitted to observe, that the oxysulphuret of antimony employed in the present formula, was also used in the Pharmacopœia of 1815: indeed, throughout his work, though constantly referring to the Pharmacopœiæ of 1720, 1745, 1788, 1809, and 1824, he makes no mention of that of 1815. The present instance, however, (where the Pharmacopœia of 1809 directs the use of oxide of antimony,) may show him that the Pharmacopœia of 1815 is by no means a mere reprint of that of 1809.

The *Vinum Antimonii Potassio-tartratis* was not called *Liquor Antimonii Tartarizati* in the last Pharmacopœia, as Mr. Phillips asserts, (p. 193,) but *Vinum Antim. Tart.* The use of sherry-wine in this preparation is now restored.

In speaking of the nitrate of silver, Dr. Spillan says, "When the patient has been for some time using this medicine, his skin acquires a black hue, which continues for a considerable time even after the medicine has been discontinued." (P. 148.) It would be much more correct to say, "his skin occasionally acquires a leaden hue, which unfortunately is permanent;" or, as Dr. Collier expresses the caution, "it should be recollected that the remedy, if long persisted in, may induce permanent discoloration of the skin." To this we would append as a corollary, that this remedy ought hardly ever to be internally administered.

The following are the remainder of the new names in this section:

| New Names. | Old Names. |
|---|---|
| Pulvis Antimonii comp. | Pulvis Antimonialis. |
| Liquor Potassæ Arsenitis, | Liquor Arsenicalis. |
| Bismuthi Trisnitras, | Bismuthi Subnitras. |
| Calcii Chloridum, | Calcis Murias. |
| Liquor Calcii Chloridi, | Liquor Calcis Muriatis. |
| Cupri Ammonio-Sulphas, | Cuprum Ammoniatum. |
| Liquor Cupri Ammonio-Sulphatis, | Liquor Cupri Ammoniati. |
| Ferri Sesquioxydum, | Ferri Subcarbonas. |
| Tinctura Ferri Sesquichloridi, | Tinctura Ferri Muriatis. |
| Ferri Potassio-Tartras, | Ferrum Tartarizatum. |
| Ferri Ammonio-Chloridum, | Ferrum Ammoniatum. |
| Tinctura Ferri Ammonio-Chloridi, | Tinctura Ferri Ammoniati. |
| Hydrargyri Bichloridum, | Hydrargyri Oxymurias. |
| Liquor Hydrargyri Bichloridi, | Liquor Hydrargyri Oxymuriatis. |
| Hydrargyri Ammonio-Chloridum, | Hydrargyrum Præcipitatum album. |
| Hydrargyri Oxydum, | Hydrargyri Oxydum cinereum. |
| Hydrargyri binoxydum, | Hydrargyri Oxydum rubrum. |
| Hydrargyri bisulphuretum, | Hydrargyri Sulphuretum rubrum. |
| Hydragyri sulphuretum cum Sulphure, | Hydrargyri Sulphuretum nigrum. |
| Magnesiæ Carbonas, | Magnesiæ Subcarbonas. |
| Liquor Plumbi Diacetatis, | Liquor Plumbi Subacetatis. |
| Liquor Plumbi Diacetatis dilutus, | Liquor Plumbi Subacetatis dilutus. |
| Potassæ Carbonas, | Potassæ Subcarbonas. |
| Liquor Potassæ Carbonatis, | Liquor Potassæ Subcarbonatis. |
| Potassæ Bicarbonas, | Potassæ Carbonas. |
| Potassæ Hydras, | Potassa fusa. |
| Potassæ Bisulphas, | Potassæ Supersulphas. |
| Potassii Sulphuretum, | Potassæ Sulphuretum. |
| Sodæ Carbonas, | Sodæ Subcarbonas. |
| Sodæ Carbonas exsiccata, | Sodæ Subcarbonas exsiccata. |
| Sodæ Sesquicarbonas, | Sodæ Carbonas. |
| Sodæ Potassio-Tartras, | Soda tartarizata. |

This table will be the more useful to our readers, inasmuch as the usual dictionary of old and new names at the end of the Pharmacopœia has been mutilated by one of its halves: the old names are given with the new synonymes, but not the new ones with the old synonymes. That useful table, moreover, giving the proportions in which opium, mercury, &c. are contained in certain preparations, has been omitted; and these defects have been supplied by none of the translators.

The formulæ for making ammoniated iron, potassio-tartrate of iron, red oxide of mercury, white precipitate of mercury, calomel, corrosive sublimate, and solution of corrosive sublimate, have all been altered. The last-mentioned formula is now without spirit, but contains hydrochlorate of ammonia, which, according to Mr. Brande, will render it less liable to decomposition. (*Manual of Pharmacy*, p. 295.)

The process given for making *Creta præparata* is merely a repetition of the old and objectionable one, and is hardly on a par with the other improvements in pharmaceutical chemistry. We understand that a very pure and much more elegant preparation—a real carbonate of lime, in fact,—is to be met with in some shops. It is obtained by decomposing a solution of muriate of lime by the carbonate of potash or soda.

*Misturæ.* Three new mixtures have been added, the *Mist. Cascarill Comp.; Mist. Gentianæ Comp.;* and *Mist. Spiritus Vini Gallici.*

The *Mist. Cornu usti* has been thrown out; the *Mist. Acaciæ* is the old *Mucilago Acaciæ.*

*Olea.* One oil has been added, the *Oleum Sambuci.*

*Pilulæ.* Six new pills have been added; namely, the *Pil. Conii Comp.; Pil. Hydrargyri Iodidi; Pil. Ipecacuanhæ Comp.; Pil. Rhei Comp.; Pil. Sagapeni Comp.;* and *Pil. Styracis Comp.*

The *Pil. Hydrargyri Submuriatis Comp.* are now called *Pil. Hydrargyri Chloridi Comp.;* and the *Pil. Saponis cum Opio* are now called *Pil. Saponis Comp.;* probably for the sake of concealing the active drug from patients who pry into prescriptions. The *Pil. Styracis Comp.*, which contain opium in the same proportion, will serve the same purpose. The following is Dr. Collier's manner of informing his readers whence this formula is derived: "This they take word for word from the Dublin, as they have, in the course of time, taken every other formula, bit by bit, at the expense of general industry, and then say they, 'Behold our copy-right!'" (P. 195.)

In the formula for the *Pil. Hydr. Chloridi Comp.*, (Plummer's pill,) treacle is directed to be used instead of spirit. In the Pharmacopœia of 1809, copaiba was employed as in Plummer's original form, (*Edin. Med. Essays*, vol. I. p. 59;) but, in that of 1815, it was changed for mucilage of acacia. It is curious that this alteration escaped the observation of Dr. Thomas Young, who says, "The copaiba employed for forming these pills ought to be changed for gum, since it envelopes the substances so much as to lessen their activity. The subcarbonate of iron, made up into pills with copaiba, was given for some weeks without any apparent effect; and a few hours after the same quantity had been given with gum only, the fæces were perfectly black." (*Med. Liter.* p. 474, 2d Edit. 1823.) In 1824, the gum was changed for spirit, and this again was objected to. A correspondent of the Medical Gazette complains of the pills passing through the bowels undissolved, and suggests giving the substances which compose Plummer's pill, in the state of powder. He complains of the copaiba as well as the spirit, and, like others, has overlooked the gum in the Pharmacopœia of 1815. (*Med. Gaz.*, vol. II. p. 110.)

We once knew an instance where refractory pills of this sort, taken by an old gentleman for a chronic disease of the skin, led to the curious mistake of the patient being considered by his apothecary (one of the old school) as affected with *gall-stones.* We found the supposed calculi carefully preserved, literally in boxfuls, and we believe in the very boxes from which they had been originally taken!

The *Pil. Rhei Comp.* are aromatized, not with oil of peppermint as in the Edinburgh Pharmacopœia, but with oil of carraway. This will in some cases superinduce the advantage, or disadvantage, of making a familiar pill strange to the patient.

*Pulveres.* One new powder has been added, the *Pulv. Jalapæ Comp.;* three old ones have been expunged, namely, the *Pulv. Cornu usti cum Opio, Pulv. Contrajervæ Comp.*, and *Pulv. Sennæ Comp.* Let us take this opportunity of mentioning the singular fact, that Dover prescribed his powder in doses of from forty to seventy grains. Dr. Young indeed mentions it, (*Med. Liter.*, 2d Edit. p. 502,) but it is by no means generally known. It is not in chronic rheumatism, as one might have conjectured, but in gout that Dover recommends it; he says, "Dose from forty to sixty or seventy grains in a glass of white wine posset, going to bed. Covering up warm, and drinking a quart or three pints of the posset drink while sweating." (*The ancient Physician's Legacy to his Country*, p. 18-19.) Could the largest of these doses be safely given? And, if so, are we to conclude that the narcotic power of opium is diminished one half by combining it with ipecacuanha, or that opium uncombined may be given in these terrifying doses?

*Spiritus.* Alcohol is now made by distilling rectified spirits with chloride of calcium (muriate of lime,) instead of subcarbonate (carbonate) of potash. There are no new spirits, nor have any old ones been expunged, but several have been transferred, with great propriety, to the tinctures, as they were made by maceration, not distillation. Strange to say, the list of these changes at the end of the Pharmacopœia contains only two out of four; we will therefore give our readers a complete one.

| Old Names. | New Names. |
|---|---|
| Spir. Ammoniæ succinatus, | Tr. Ammoniæ Comp. |
| Spir. Camphoræ, | Tr. Camphoræ. |
| Spir. Colchici ammoniatus, | Tr. Colchici Comp. |
| Spir. Lavandulæ Comp. | Tr. Lavandulæ Comp. |

The *Tr. Ammon. Comp.* (*Eau de Luce,*) is now made with *Liq. Ammon. fortior*, instead of *Liq. Ammon.;* we must therefore warn our readers to diminish their doses.

*Syrupi.* The *Syr. simplex* is now called merely *Syrupus*, and the *Syrupus Aurantiorum* is called *Syrupus Aurantii.* The following remarks by Dr. Collier, on the adulteration of *Syrupus Papaveris*, are important, and deserve the attention of all practitioners.

"There is a bad practice in the retail trade (which is well worthy of the serious attention of the profession, and one which ought not to have been disregarded by the College) of selling various substitutes for this syrup. One is prepared with laudanum and treacle; another, with extract of poppies in syrup; and both made of inferior narcotic strength to the preceding. Now, let the dispensing chemist mark the consequence. Numerous fatal cases have occurred in which mothers, who had been in the habit of being supplied with the spurious syrup, have casually applied to houses where the syrup is prepared by the College formula; and, having administered a teaspoonful of this last to their fretful children, or, perhaps, a second, just as they were wont to do with the weaker remedy, it has induced narcosis, and death has ensued in a few hours. The Editor has himself attended inquests of this nature, and he appeals to the Coroners of London, whether a year elapses without similar occurrences. The intention of the dispensing chemist in making a weaker article is praiseworthy; for, knowing that mothers habitually exhibit it to their children, he is afraid to sell them the stronger syrup. Thus the parent is deceived in her estimation of dose, and the deception is fatal. These errors, too, are likely to occur among practitioners themselves; for, if they consult the popular works on pharmacy, they will meet with evidence the most discrepant. By one author an ounce of the syrup is stated to be equivalent to a grain of opium; by another, half an ounce; by another, three drachms; by another, two drachms." (P. 209.)

*Tincturæ.* The following new tinctures have been added: *Tr. Balsami Tolutani; Tr. Colchici; Tr. Conii; Tr. Cubebæ; Tr. Gallæ; Tr. Iodinii Comp.*

The *Tr. Cinchonæ* is now made with the heart-leaved bark, but the *Tr. Cinchonæ Comp.* is still made with the lance-leaved species.

The *Tr. Cinchonæ ammoniata* has been omitted, and also the *Tr. Rhei;* the *Tr. Rhei Comp.*, however, has been retained and somewhat altered. The proportion of rhubarb has been slightly diminished, and proof spirit alone is used, without the addition of water. In the *Tr. Camphoræ Comp.* the oil of aniseed, omitted in the Pharmacopœiæ of 1809, 1815, and 1824, has been restored. The *Tr. Digitalis* is rather weaker, as four ounces of the leaves are now used to two Imperial instead of wine pints.

It is remarkable that the Tincture of Opium is directed to be made with proof-spirit in the octavo, and with rectified spirit in the pocket edition. As Mr. Phillips gives proof spirit in his translation, and this is also the direction of the Pharmacopœiæ of 1809, 1815, and 1824, we must conclude that the error is in the small edition. Dr. Spillan retains the reading of the small edition. Dr. Collier adopts Mr. Phillips's reading, animadverting, however, on the error of the small edition in his peculiar style. "In the College text they have 'rectificati,' but, when they commit an error, they follow it up with silent dignity." (P. 228.)

*Vina.* These are now prepared with Sherry.

The *Vinum Aloës* is much weaker, containing one part of aloes to twenty of fluid, instead of one to eight. The *Vinum Colchici* is now prepared with the dried instead of the fresh cormus, and therefore the proportion of the drug has been reduced to one-fifth of the former one. There are now six preparations of colchicum in the Pharmacopœia; namely, a watery extract, an acetic extract, a vinegar, two tinctures, (both made with the seeds,) and a wine.

*Unguenta.* Eight ointments have been added; the *Ung. Antimonii Potassio-Tartratis; Ung. Creasoti; Ung. Gallæ Comp.; Ung. Hydrargyri Iodidi; Ung. Hydrargyri Biniodidi; Ung. Iodinii Comp.; Ung. Plumbi Comp.;* and *Ung. Plumbi Iodidi.*

The first on this list, the Tartar-Emetic ointment, is, we think, stronger than it is generally prescribed, as it contains one part of the salt to four of lard. We believe the usual strength to be one part of tartar emetic to eight of lard, as we find it in the Pharmacopœia of St. George's Hospital, (*London*, 1816.) These, too, were the proportions commonly employed by Jenner. (*On the Influence of Artificial Eruptions. London*, 1822.) He remarks, however, that it is sometimes necessary to make it stronger, and gives the following formula for such cases:—℞. Antim Tartrat. (subtil. pulv.,) ʒii.; Ung. Cetacei, ʒix.; Sacchari Albi, ʒi.; Hydrar. Sph. Rub., gr. v.; M. fiat Unguentum. He says that sugar prevents the ointment from becoming rancid.

"A patient applied the ointment according to the preceding formula, at night, and had eruptions next morning, which was within a space of twelve hours. He had, however, used the same on a preceding occasion, in cold weather, and with a skin less perspirable: in this instance it was much tardier in vesicating." . . . "In the case of a lady, where two parts of tartar emetic and one of simple cerate were used, eruptions appeared in a few hours." (P.54.)

We once met, in dispensary practice, with a singular case of what Dr. Jenner calls torpor of the skin. A man of about sixty having applied a blister to his chest without effect, we rubbed the potassa fusa on his skin, but it was equally ineffective. We had frequently seen blisters powerless, whether from the badness of the cantharides, from the patient heating the plaster too much, or from the plaster not having been really applied; but this is the only case in which potassa fusa (and it was of excellent quality,) has proved inefficient in our experience.

The *Ung. Creasoti* is intended, as we learn from Dr. Collier and Mr. Phillips, for slight cases of ringworm: and Dr. Spillan says it has been successfully used in the treatment of ulcers and burns.

The *Ung. Gallæ Comp.* differs from the *Ung. Gallæ* of the Edinburgh Pharmacopœia by the addition of opium.

The *Ung. Hydrargyri Iodidi* and *Ung. Hydrargyri Biniodidi* "have been used, chiefly in hospital practice, for ill-conditioned sores which had long resisted the ordinary preparations of mercury, and also for scrofulous ulcers." (*Collier*, p. 240.) They were first introduced for the treatment of syphilis by the French; but the London ointments contain the mercurial salt in the proportion of 1 to 8, while Magendie's have only twenty grains of the iodide or biniodide of mercury to an ounce and a half of lard. (*Formulaire*, 8e Edit. p. 249.)

The *Ung. Iodinii Comp.* is to be used in bronchocele; the *Ung. Plumbi Comp.* for indolent ulcers; and the *Ung. Plumbi Iodidi*, say the translators, is employed in chronic enlargements of the joints, and other scrofulous tumours.

Oil of Bergamot has been added both to the simple and the compound sulphur ointment.

Such are the more prominent points of difference between the late and the present Pharmacopœia. We have given them with a minuteness which will perhaps to some appear superfluous. But the collation which we have gone through is what few, even with the new and old publications in their hands, would take the trouble of making; and we trust, therefore, rather to receive thanks for our industry, than a rebuke for our minuteness.

---

